# Antimicrobial resistance pattern of *Klebsiella* isolated from various clinical samples in Ethiopia: a systematic review and meta-analysis

**DOI:** 10.1186/s12879-023-08633-x

**Published:** 2023-10-02

**Authors:** Leake Gebremeskel, Tewolde Teklu, Gebremicheal Gebreslassie Kasahun, Kald Beshir Tuem

**Affiliations:** 1 Department of Pharmacology and Toxicology, School of Pharmacy, College of Health Sciences, Mekelle University, Mekelle, Ethiopia; 2 Department of Pharmacy, College of health sciences, Aksum University, Aksum, Ethiopia

**Keywords:** *Klebsiella* resistance, Antimicrobial resistance, Meta-analysis, Systematic review, Ethiopia

## Abstract

**Background:**

The burden of *Klebsiella* drug resistance to antimicrobials is a major public health concern worldwide; particularly the problem is severe in developing countries including Ethiopia. Therefore, the aim of this systematic review and meta-analysis is to establish the pooled estimate of *Klebsiella* drug resistance; and antimicrobial-specific resistance pattern among *Klebsiella* clinical isoaltes in Ethiopia.

**Methods:**

Articles were searched from PubMed, Google Scholar, and Science direct and grey literature from 2009 to 2019. Four authors have independently extracted data on the prevalence and antimicrobial resistance pattern of the isolates. Statistical analysis was conducted by using Open meta-analyst (version 3.13) and Comprehensive meta-analysis (version 3.3). The main outcome measures were the overall *Klebsiella* resistance; and drug-specific resistance patterns. A random-effects model was used to determine the pooled resistance prevalence with 95% confidence interval (CI), and significant heterogeneity was considered at p < 0.1; and *I*^2^ > 50% using DerSimonian and Laird method. In addition, subgroup analyses were conducted to improve the outcome.

**Result:**

We obtained 174 potentially relevant studies through searching electronic databases, and finally, 35 eligible studies were included for meta-analysis. A total of 13,269 study samples participated, from which 1017 *Klebsiella* species were isolated. The overall *Klebsiella* resistance in Ethiopia was found to stand at 53.75% (95% CI: 48.35—58.94%). Based on the subgroup analyses; the highest (64.39%); and lowest (46.16%) values were seen in Southern Nations, Nationalities, and Peoples of Ethiopia; and Tigray regions respectively; and the highest *Klebsiella* resistance was reported to ampicillin (90.56%), followed by amoxicillin (76.01%) and trimethoprim-sulfamethoxazole (66.91%). A relatively low level of resistance rate was observed to amikacin (16.74%) and cefoxitin (29.73%).

**Conclusion:**

The pooled *Klebsiella* resistance was found to be considerably high (53.75%) to most of the essential antibiotics in Ethiopia. *Klebsiella* was highly resistant to ampicillin and amoxicillin but relatively lower to amikacin. Therefore, appropriate interventional strategies need to be taken to address the emerging resistance of *Klebsiella* species.

**Supplementary Information:**

The online version contains supplementary material available at 10.1186/s12879-023-08633-x.

## Background

*Klebsiella* species are aerobic or facultative anaerobic gram negative bacteria which belong to the family *Enterobacteriaceae*. It is known to produce plasmid mediated extended spectrum beta lactamases (ESBLs) which breaks down antibiotics into inactive form [1] and the most common isolated species include *Klebsiella pneumoniae, Klebsiella oxytoca, Klebsiella ozaenae and Klebsiella rhinoscleromatis* [2, 3]. From the various species, *Klebsiella pneumoniae* is the most common and ubiquitous in nature causing various human infections [4, 5].

The widespread use of antimicrobials in clinical practice has led to the emergence of resistant bacterial pathogens contributing to the increased morbidity and mortality observed worldwide [6, 7]. Resistant *Klebsiella* is one of the opportunistic pathogen showing frequent acquisition of resistance to antibiotics accounting to about one-third of all Gram-negative infectious diseases [8, 9] such as bloodstream infection, pneumonia, urinary tract infections (UTI), nosocomial and community acquired infections [2, 10]. As there is inappropriate use of antimicrobials, resistance is tremendously increasing and the therapeutic option has been significantly reduced [11, 12]. Ultimately, this increases cost of treatment and impedes the effective prevention and treatment outcomes in clinical settings [13–16].

Nowadays, studies show that *Klebsiella* has been resistant to most common antibiotics including cephalosporins, monobactams, fluoroquinolones and aminoglycosides [3, 17–19]. This is because of the frequent empirical use of antibiotics; and persistent exposure of *Klebsiella* to a number of antimicrobial agents which facilitate the emergence of drug-resistant strains [20]. Previous studies have indicated *Klebsiella’s* resistance to antibiotics reaching 68.3% in south Africa [12], 54% in India [1], and 97.17% in Equatorial Guinea [21]. In sub-Saharan Africa including Ethiopia, the anti-microbial drug resistance is a serious problem [22]. Individual studies conducted in Ethiopia showed that the prevalence of antimicrobial resistance is high and pooled prevalence of resistance in gram negative bacteria *Klebsiella* pneumonia is reported to be 23.2% [23]; *Klebsiella* species are able to develop cross resistance; and the treatment failure is also high [3]. Moreover, one study indicates that antimicrobial resistance level of the gram-negative bacteria ranges from 20 to 100% [24]. Studies in some Ethiopian hospitals such as Jimma (77.8%) [25], Yekatit 12 (64.7%) [26] and Gondar hospitals (95.6%) [27], show that the antimicrobial resistance of *Klebsiella* is high. However, the comprhenssive analysis of antimicrobial drug resistance pattern of *Klebsiella* isolates from different parts of Ethiopia has not yet been performed nationally. Hence, the present systematic review and meta analysis was aimed at establishing the pooled prevalence of *Klebsiella* resistamce; and antimicrobial-specific resistance pattern among *Klebsiella* clinical isolates in Ethiopia.

## Methods

This meta-analysis was carried out in a similar approach to the previously published studies [13, 28] and the content of this systematic review and meta-analysis is well described according to the Preferred Reporting Items for Systematic Reviews and Meta-Analysis (PRISMA) flow diagram and checklist [29–31] (S1 file).

### Study selection

Exhaustive literature search was done systematically in PubMed, and Science direct, Google scholar; and manual search from Google of potentially relevant articles. The search was done by four authors (LG, TT, GGK and KBT) independently; and the search strategy was built by combining the three main arms (Table 1): *Klebsiella*, Antimicrobial related terms, and Ethiopia. From the citation extracted, abstracts were scanned to retrieve the clinical studies on *Klebsiella* infection. Studies that were relevant, by title and abstract, were accessed in full text to determine articles to be included in our meta-analysis. Finally, the references cited by each eligible study were scrutinized to identify additional source; and references were cited using endnote citation manager software (version X7)[Niles software, Clarivate analytic company]. Before starting the data extraction, the protocol was developed and we went directly to data extraction without considering the registration, because at that time there was internet interruption because of the conflict here in Tigray, Ethiopia.

**Table 1:** Search arms and terms used as a searching strategy

Search arms	Antimicrobial resistance related keywords (A)	*Klebsiella* related keywords (B)	Ethiopia (C)
**Search terms**	“drug resistance” OR “antibiotic resistance” OR “antimicrobial resistance” OR “antimicrobial drug resistance” OR “anti- infective resistance” OR “Antibacterial resistance” OR “Antibiotic susceptibility*”OR “Drug susceptibility” OR “Anti-biotic sensitivity” OR Drug sensitivity”	“*Klebsiella*” OR “*Klebsiella spp*.” OR “Klebsiella*” OR ”*Klebsiella pneumoniae*” OR “*K.pneumoniae*” OR “*K.oxytoca*” OR “*k.terrigna*” OR”*k.planticola*”	Ethiopia

Search terms within each arm were combined using the Boolean term “OR” and finally “AND” was used to connect the three search arms (“Column A” AND “Column B” AND “Column C”)

### Inclusion and exclusion criteria

Studies included in this comprehensive meta-analysis were those that had extractable data on drug resistance of *Klebsiella* isolates taken from human samples in Ethiopian health facilities or research centers. Articles published from January 2009 to December 2019 in English language; and primary researches with cross-sectional study designs were included; but review papers and other studies that were not relevant to the outcomes of interest and those that were not based on Ethiopian setting were excluded. Initially, non- relevant studies were excluded based on their titles and abstracts. From the screened papers, the duplicates, incomplete data, and studies with very small number of isolates and tested antimicrobials (less than 5 isolates and tested drugs) were excluded.

### Outcome of interest

The primary outcome of interest was the prevalence of antimicrobial resistance of *Klebsiella* species among the total *Klebsiella* clinical isolates in which the prevalence was calculated by dividing the numbers of resistant *Klebsiella* isolates by the total number of clinically isolated *Klebsiella*. We also calculated the pooled resistance pattern of *Klebsiella* isolates to specific antibiotics as a secondary outcome of interest.

### Data extraction

The process of screening (by title, abstract, and full text and data extraction) was done independently by three authors (LG, GGK, and TT), and at each step KBT was involved in consensus creation in cases of discrepancies among the three authors. During screening by title and abstract, the authors reviewed the full text of the article if needed. Data from eligible studies were extracted and summarized into an excel spreadsheet. For each of the included studies, the following information was extracted; name of regions, study design, study area/city, study names, study period, study design, types of specimens, study population, number of study participants, total numbers of isolated *Klebsiella* species, the average percentage of resistant *Klebsiella* species, antimicrobial resistance rate of *Klebsiella* species and references. As all of the articles used in this study are cross-sectional, the score for the quality of the study was assessed using the modified Newcastle-Ottawa Scale (NOS)[32, 33] for the representativeness of sample, appropriateness of sample size, response rate, validity of method, strategy to control confounding factors, reliability of outcome determination, and appropriate statistical analyses. The quality score (Table 2) disagreements were resolved by consensus and a final agreed-upon rating was assigned to each study (S2 file). Moreover, from the included studies each antibacterial tested for *Klebsiella was* extracted (Table 3).

**Table 2:** Summary of 35 studies reporting the prevalence and resistance pattern of *Klebsiella* in different parts of Ethiopia, 2009–2019

References	Region	Study area	Study period	Study design	Study population	Culture specimens	Study samples	#Klebsiella isolated	Average % resistant Klebsiella	NOS quality score out of 7
[42]	Amhara	Gondar	March to May 2013	RCS	Patients	Blood sample	856	11	**46.4**	6
[43]	Amhara	Debremarkos	November 2013 to February 2017	RCS	Patients	Blood, urine	514	22	**57.8**	6
[44]	Tigray	Mekelle	August-December 2016	CS	HIV patients	Sputum	252	41	**19.6**	5
[45]	SNNP	Hawassa	November 2014–November 2017	CS	HIV positive at ART and blood doners	Urine, Body fluids, ear swab, eye swab, Blood, sputum and pus	693	154	**65.4**	6
[46]	SNNP	Hawassa	January 2012 to December2014	RCS	Patients	Urine, nasal swab, pus, and ear discharge	564	21	**63.0**	6
[47]	Oromiya	Jimma	February to August, 2016	CS	Catheterized patients	Urine sample	143	12	**62.5**	7
[48]	Central Ethiopia	Addis Ababa	December 2017-June 2018	CS	Patients	Urine,blood	947	72	**67.2**	6
[49]	Central Ethiopia	Addis Ababa	September 2015-May 2016	CS	UTI patients	Urine	712	19	**45.0**	7
[50]	Central Ethiopia	Addis Ababa	Feburary-May 2015	CS	Fistula patients	Urine	210	14	**29.8**	4
[51]	SNNP	Hawassa	Novemer 2010-March 2011	CS	Patients with surgical wound	Wound swabs	194	24	**80.4**	4
[26]	Central Ethiopia	Addis Ababa	January - April, 2014	CS	Pediatric Patients	Urine	384	17	**59.4**	6
[27]	Amhara	Gondar	Feburary-March, 2014	CS	UTI patients	Urine	442	34	**40.9**	6
[52]	Amhara	Gondar	February to May, 2016.	CS	Patients	stool, urine, pus, eye discharge and body fluids	260	29	**52.8**	5
[53]	Oromiya	Jimma	May to September,2016	CS	Hospitalized patients	urine, blood, sputum samples	240	30	**70.9**	7
[54]	Tigray	Mekelle	February to September, 2017	CS	UTI patients	urine samples	341	6	**43.3**	4
[55]	Amhara	Gondar	January to April, 2016	CS	Patients with Ocular infection	Conjunctival specimens	312	9	**38.4**	4
[56]	Oromiya	Jimma	June to December 2011	CS	Patients with wound	wound discharge swabs	322	46	**43.5**	6
[57]	Oromiya	Metu	January to March,2018	CS	Infected DM patients,	Urine	233	8	**26.6**	6
[58]	Amhara	Bahridar	January 2013 to December 2015	RCS	Infected Wounds	Wound swabs	380	20	**43.1**	7
[59]	Central Ethiopia	Addis Ababa	January to March,2014	CS	Children with UTI and sepsis	Blood,and Urine	322	20	**69.0**	7
[60]	Oromiya	Jimma	May to September, 2013	CS	Patients with wound	wound discharge swabs	150	14	**63.3**	6
[61]	Central Ethiopia	Addis Ababa	August 2013 to January 2014	CS	UTI patients	urine samples	424	7	**55.8**	4
[62]	Tigray	Mekelle	January to June 2012	CS	Patients with surgical wound	Wound swabs	610	29	**71.5**	5
[63]	SNNP	Hawasa	May to September 2015	CS	UTI patients	urine samples	863	18	**51.2**	6
[64]	Amhara	Bahridar	December 2017 to April 2018	CS	Patients	blood, urine, ear discharge,	532	85	**73.2**	5
[6]	Amhara	Gondar	March to May, 2014	CS	Wound patients	Wound discharge	137	17	**62.1**	6
[65]	Amhara	Gondar	January to June 2017	CS	Patients with otitis media	ear discharge	62	10	**31.1**	5
[66]	Central Ethiopia	Addis Ababa	October 2011 to Feburary 2012	CS	Septicemia suspected children	Blood	201	9	**62.9**	7
[67]	Oromiya	Jimma	June2012 to Feburary 2013	CS	Patients with postsurgical infection	Urine and wound swab	500	12	**54.6**	6
[68]	Oromiya	Assela	April 2016 to May 2017	CS	Neonatal ICU patients	Blood sample	303	11	**63.9**	6
[69]	SNNP	Hawassa	April to July 2018	CS	Pediatrics with otitis media	Ear discharge	152	12	**54.6**	6
(70)	Tigray	Adigrat	January to April 2018	CS	Pregnant women	Urine sample	259	10	**35.8**	7
(71)	Central Ethiopia	Addis Ababa	January to May 2017	CS	Patients	Urine, blood, sputum, ear discharge, CSF, body fluid, Pus	426	131	**52.6**	7
(72)	Tigray	Mekelle	October 2014 to June 2012	CS	Patients with otitis media	Ear discharge	162	18	**60.4**	6
(73)	Amhara	Gondar	Feburary to June 2014	CS	Patients with ear discharge	Ear discharge	167	25	**44.6**	6

Key: CS: Cross-sectional, RCS: retrospective cross-sectional, CSF: cerebrospinal fluid, ART: Antiretroviral therapy, HIV: human immunodeficiency virus

**Table 3:** Percentage of pooled antibiotic resistance rates of *Klebsiella*; Ethiopia, 2009–2019

Antibacterial (%)																	
Studies	AMP	AML	AMC	CN	CIP	NOR	C	TE	SXT	AMK	CRO	NA	F	CAZ	CFP	FOX	CTX
Abebaw et al., 2018 [42]	72.7	72.7		45.4	54.5	9.1		72.7	63.6		36.4	9.1				27.8	
Abebe et al.,2019 [43]	100	59	40.9	46	41	50	46	81.8	77		36.0						
Adhanom et al.,2019 [44]				14.6	2.4		7.3	19.5	29.3	9.7	7.3		66.7				
Alemayehu et al.,2019 [45]	93.8		74.4	79.3	41.3	43.8	51	100	91.5		86.6		7.1	51			
Amsalu et al.,2017 [46]	100		37.5	71.4	33.3	50	75		89		47.6						
Awoke et al.,2019 [47]	100	100	66.7	75	33.3			66.7	75	8.3	58.3		41.7				
Beyene et al., 2019 [48]			70.9	63.9	51.3	52		79.2	88.9	12.5	83.3		54.2	83.3	83.3		83.33
Bitew et al., 2017 [49]	100		21	21	15.8			42.1	63.2		42.10		57.9	42.1			
Dereje et al., 2017 [50]			0	50	78.6		28.6				14.3		7.1				
Dessalegn et al., 2014 [51]	87.5		50	100	62.5		87.5		87.5		87.5						
Duffa et al., 2018 [26]			70.6	82.4	29.4	35.3	58.3		88.2			52.9	29.4	88.2			88.2
Eshetie et al.,2015 [27]	94.1		64.7	58.8	8.8		67.5	55.9	55.9		14.7	26.5		58.8	11.8		
Feleke et al.,2018 [52]		75.9	55.2	37.9	13.8		31	44.8	48.3	17.2	48.3		55.2				
Gashaw et al.,2018 [53]	100		96.7	70	40		66.7	90	80		53.3			56.7	50	76.7	
Gebremariam et al.,2019 [54]	83.3	66.7		33.3		16.7	16.7	50	66.7		16.7	33.3	50				
Getahun et al., 2017 [55]	100	100	11.1	33.3	22.2	28.3	44.4	33.3	33.3		33.3			44.4	22.2	22.2	
Godebo et al.,2013 [56]	69.6			28.3	28.3	28.3	69.6		65.1		28.3						30.4
Gutema et al., 2018 [57]			12.5	12.5	37.5	37.5		75			12.5		25				
Hailu et al.,2016 [58]	75		50	61.1	20		44.4		40		11.1						
Legese et al.,2017 [59]		95	90	85		15	55	75	85				50			40	100
Mama et al.,2014 [60]	100			64	35.7	21	85	57	85.7		71	50					
Mamuye 2016 [61]	85.7	71.4		28.6	57.1	71.4	57.1	28.6	57.1		71.4	42.9	42.9				
Mengesha et al.,2014 [62]	89.7	100	65.5	27.8	37.9			93.1			86.2						
Mitiku et al.,2018 [63]	88.9	77.8		66.7	44.4	11.1			94.4		38.9		22.2				16.7
Moges et al.,2019 [64]			74.1	81.2	25.9		63.5	90.6	96.5		88.2			97.6	94.1	20	
Mohammed et al.,2017 [6]	94.1			29.4	58.8		70.6	64.7	64.5		52.9						
Molla et al.,2019 [65]	90		60	10			10	10	40		50	10			50		
Negussie et al., 2015 [66]	100			100	44.4		44.4	55.5	77.8		100	44.4				0	
Sahile et al., 2016 [67]	58.33		91.7	66.7	50	50	33.3	41.7	58.3				41.7				
Sorsa et al., 2019 [68]	91			82	27		56		73	36							82
Tadesse et al., 2019 [69]	100		66.7	66.7	0		50	50	41.7		66.7			50			
Tadesse et al.,2018 [70]	100		10	30.3	30				30		10		40				
Teklu et al., 2019 [71]		17.6	78.6	62.6	48.1	47.7			80.9		44.4			78.6	77.1	21.4	77.1
Wasihun & Zemen, 2015 [72]	88.9		72.2	38.9	11	50		88.9	77.8				55.6				
Worku et al.,2017 [73]	92		92	12	4		44		36		32						
**Pooled** ***Klebsiella*** **resistance**	**90.6**	**76.0**	**56.9**	**55.3**	**34.0**	**36.3**	**50.5**	**61.1**	**66.9**	**16.7**	**47.6**	**33.6**	**40.4**	**65.1**	**55.5**	**29.7**	**68.2**

**Key**: AMC: amoxicillin-clavulanic acid, AML: amoxicillin, AMP: ampicillin, C: chloramphenicol, CIP: ciprofoxacin, GEN: gentamicin, CRO: cefriaxone, F: nitrofurantoin, NA: nalidixic acid, NOR: norfoxacin, SXT: trimethoprim/sulfamethoxazole, TE: tetracycline, --: not don

### Quality control

The quality of the included studies was checked independently by two authors (LG and KBT) using a set of predetermined criteria such as research design quality of paper, completeness of extractable information, and employed methods for *Klebsiella* species isolation.

### Data analysis

A random-effects analysis method was employed to determine the pooled prevalence, subgroup analysis, and 95% confidence interval (CI) using the approach of Der- Simonian and Laird [34]. Variances and CIs were stabilized using Freeman-Tukey arc-sine methodology [35]. Heterogeneity of study results was assessed using *I*^2^ test and significant heterogeneity was considered at p < 0.10 and *I*^2^ > 50 [34, 36]. Open Meta-Analyst (version 3.13) and Comprehensive Meta-Analysis (version 3.1) statistical analyses were used. Moreover, subgroup analyses based on administrative regions and mechanism of antibacterial action were also performed to improve the specificity of the assessment of the tested drugs.

## Result

**Fig. 1:**
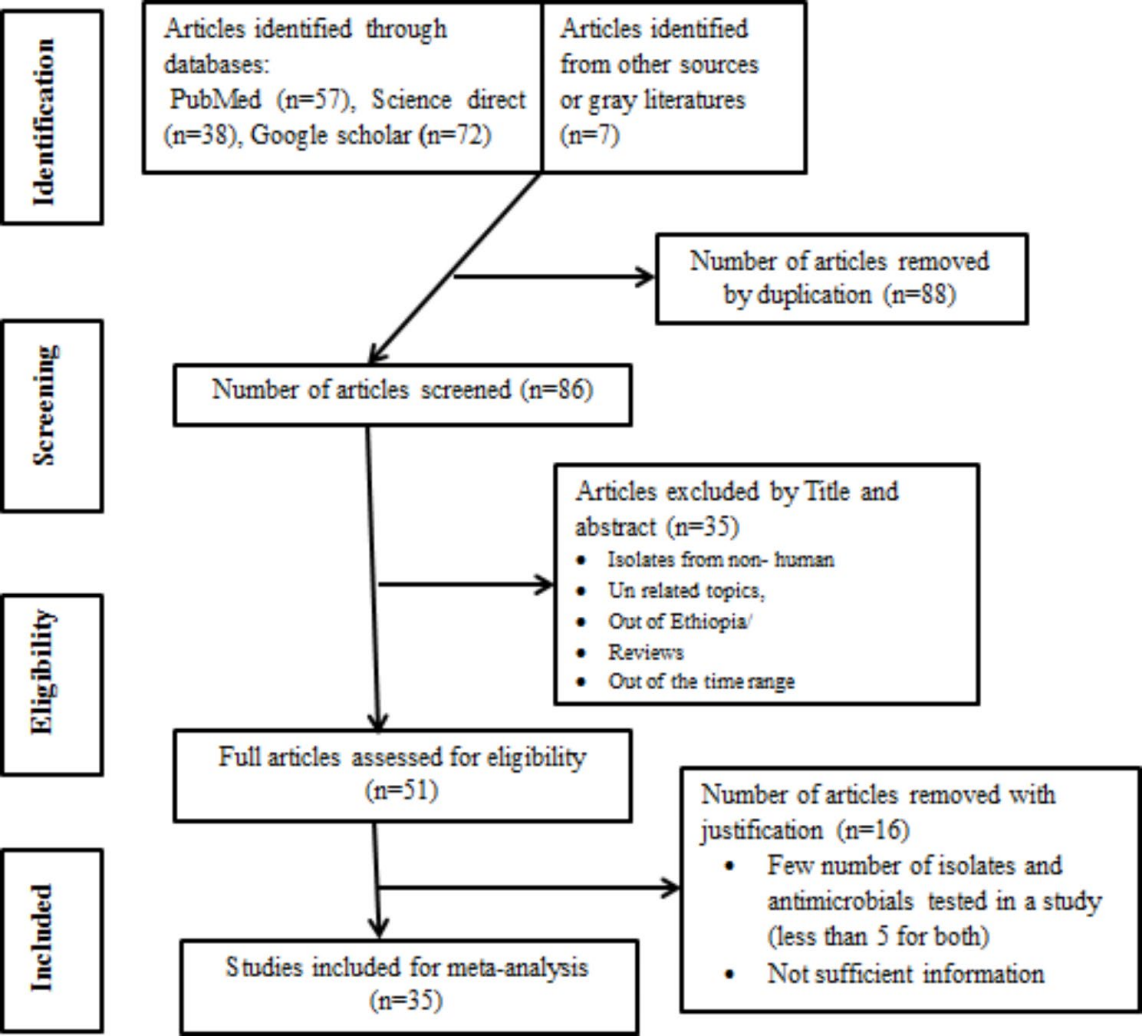
PRISMA flow diagram depicting the selection process for meta-analysis

We obtained 174 potentially relevant studies through searching electronic databases. From these, 88 duplicate articles were removed by the help of Endnote(version X7)[Niles software, Clarivate analytic company); and the remaining 86 records were screened using their title and abstract out of which 35 articles were omitted based on the exclusion criteria. Full texts of 51 records were evaluated for eligibility in which 16 articles were removed due to data incompleteness, small number of isolates and/or tested antibiotics (less than 5 *Klebsiella* isolates and antibiotics in a single study). Finally 35 eligible studies were included for the meta-analysis (Fig. 1).

Cross-sectional study design was used in all the included 35 studies. A total of 13,269 study samples were employed, from which 1017 *Klebsiella* species were isolated. The included studies were taken from Amhara, Oromia. Southern Nations, Nationalities and People (SNNP), Tigray and Addis Ababa; however, there was no study obtained from other regions of Ethiopia (Afar, Benishangul-Gumuz, Gambella, Somali, Harari and Dire Dawa city administration). For screening of *Klebsiella* species, various specimens were utilized from the various part of the human body, including blood, urine, sputum, body fluids, ear discharge, wound swab, eye swab, stools and pus (Table 2).

**Fig. 2:**
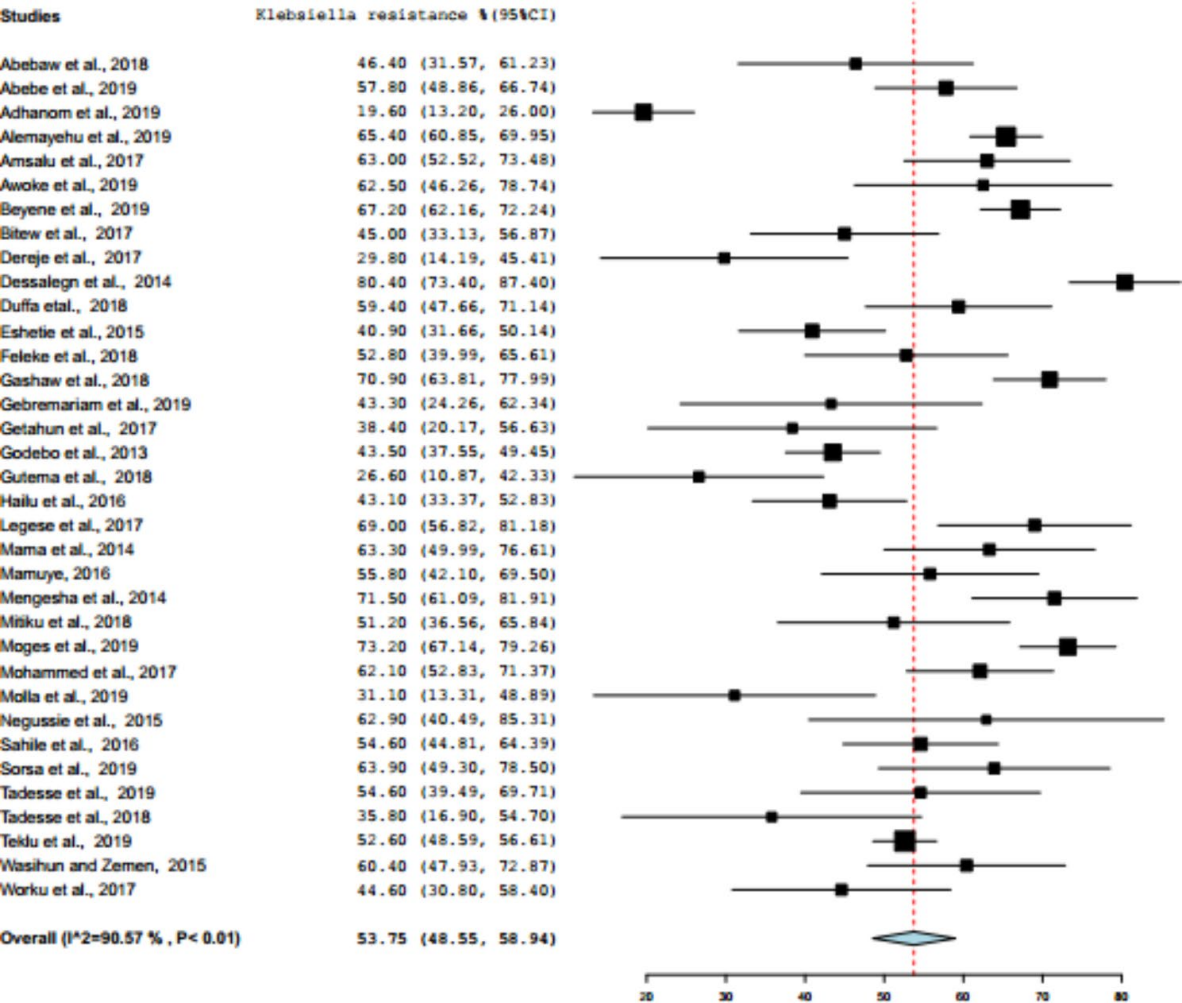
Forest plot of pooled percentage of *Klebsiella* antimicrobial resistance in 35 studies, Ethiopia, 2009–2019

**Fig. 3:**
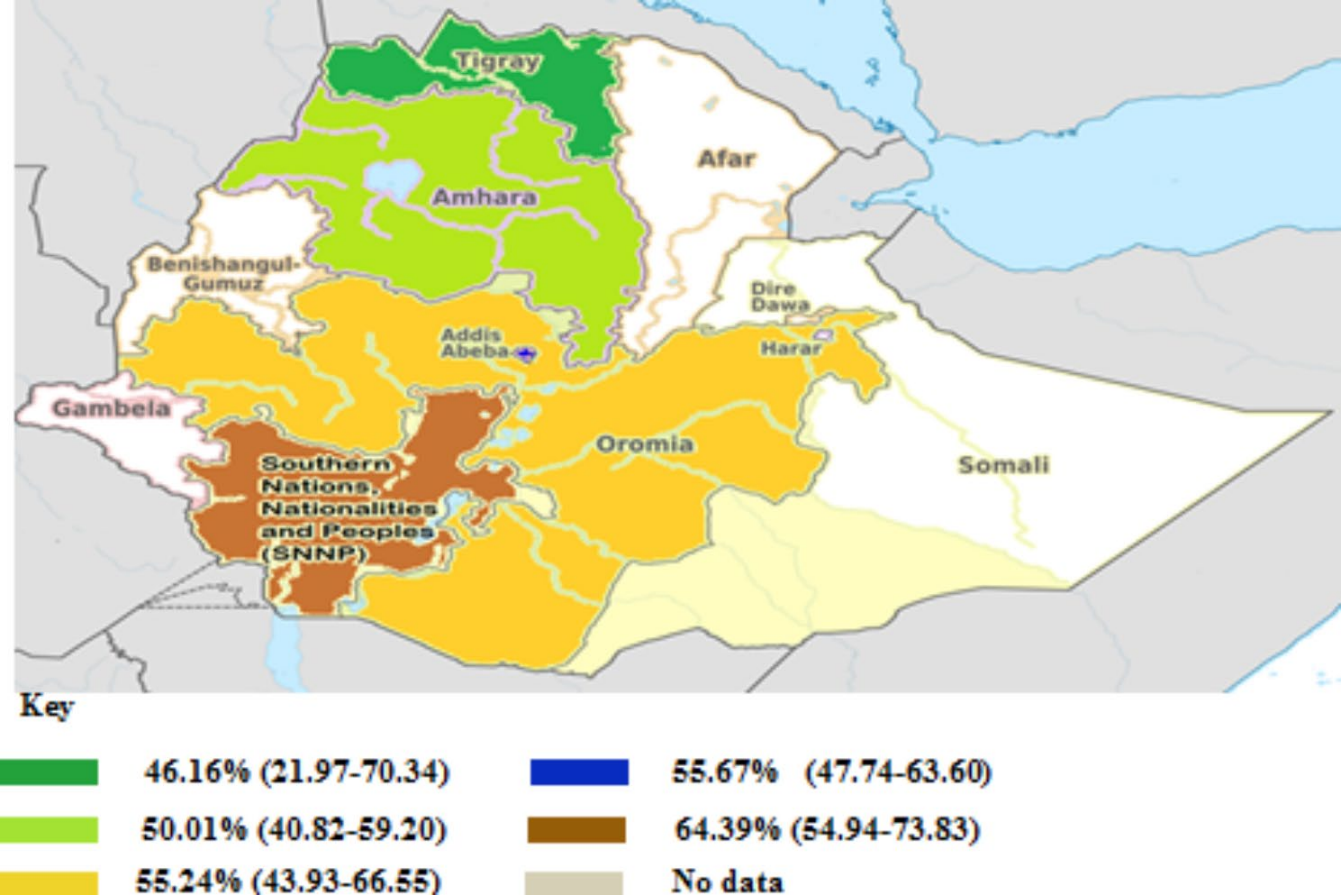
Proportion of *Klebsiella* resistance in diferent regions of Ethiopia, 2009–2019. Values in parenthesis indicated 95% CI of *Klebsiella* resistance in diferent regions of Ethiopia

**Fig. 4:**
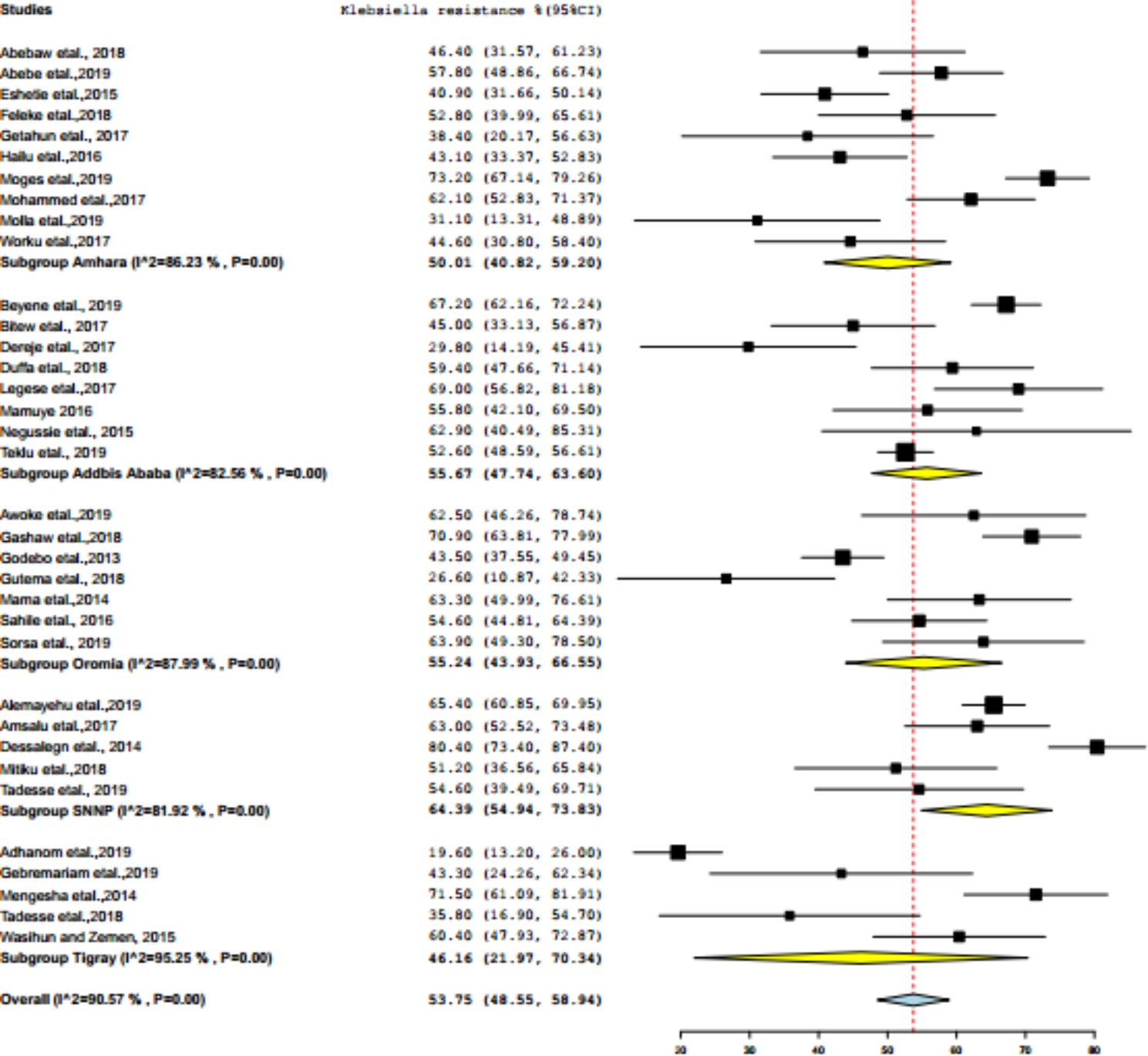
Subgroup analysis of Klebsiella antibacterial resistance according to regions of Ethiopia

The paper-based analysis of this study showed that the overall *Klebsiella* resistance in Ethiopia was 53.75% (95% CI: 48.35—58.94%) (Fig. 2). Subgroup analyses were carried out based on the region (Addis Ababa, Amhara, Oromia, SNNP, and Tigray), and then the average prevalence of *Klebsiella* resistance was determined in region wise. Southern Nations, Nationalities, and Peoples of Ethiopia was ranked first (64.39%, 95% CI: 54.94–73.83%), followed by Addis Ababa (55.67%, 95% CI: 47.74–63.40%), Oromia (55.24%, 95% CI: 43.95–66.50%), Amhara (50.1%, 95% CI: 40.82–59.20%), whereas relatively low (but highly varied) prevalence of *Klebsiella* resistance was reported from Tigray region (46.16%, 95% CI: 21.97–70.34%) (Figs. 3 and 4). The level of heterogeneity of the included studies was high, by random model methods (I^2^ = 90.55%; P < 0.01).

**Fig. 5:**
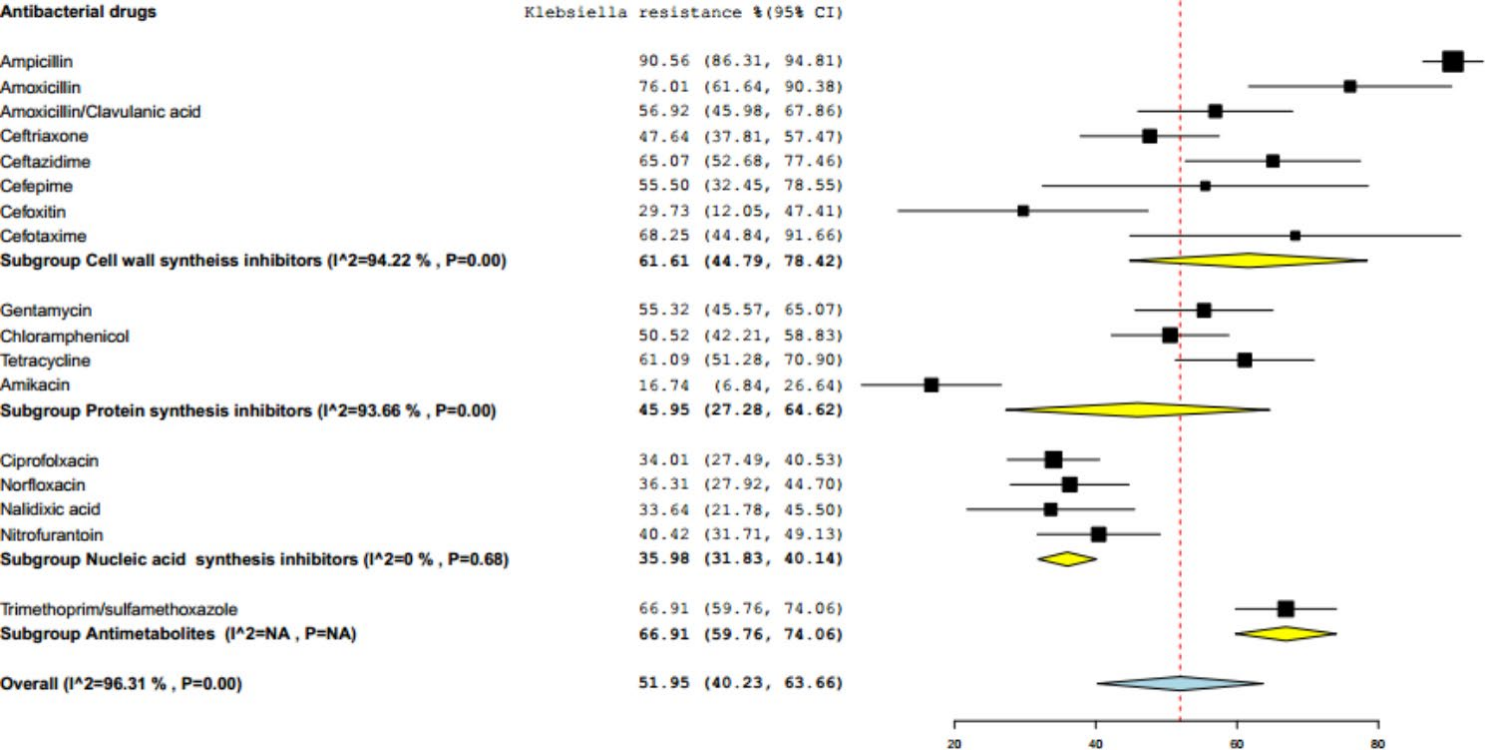
Subgroup analysis of pooled percentage and confidence interval of *Klebsiella* resistance to antibacterial drugs according to drug mechanism of action

Additional subgroup analyses were done to estimate the pooled prevalence of *Klebsiella* resistance based on the mechanism of action of the antibacterial drugs. Antimetabolite (a single drug, trimethoprim-sulfamethoxazole) accounted the highest resistance percentage (66.91%, 95% CI: 59.79–74.06%), followed by cell wall synthesis inhibitors (61.61%, 95% CI: 44.79–79.42%), protein synthesis inhibitors (45.95%, 95% CI: 27.29–64.62%), whereas nucleic acid synthesis inhibitors showed relatively low resistance percentage (35.98%, 95% CI: 31.83–40.14%) and level of heterogeneity of this class was not significant (I^2^ = 0%, p = 0.68). With regarding to individual antibiotics, high resistance rates were observed to ampicillin (90.56%, 95% CI: 86.31–94.81%), followed by amoxicillin (76.01%, 95% CI: 61.44–90.38%), trimethoprim-sulfamethoxazole (66.91%, 95% CI: 59.76–74.06%), ceftazidime (65.07%, 95% CI: 52.68–77.46%). A relatively low level of resistance rate was observed to amikacin (16.74, 95% CI: 6.84–26.64), and cefoxitin 29.73%, 95% CI: 12.05–47.41%) (Fig. 5).

## Discussion

The nationwide meta-analysis of *Klebsiella* resistance was not done in Ethiopia so far. So we conducted a comprehensive systematic review and meta-analysis to determine the pooled of *Klebsiella* antimicrobial resistance in Ethiopia. From our findings *Klebsiella* displayed diverse resistance patterns, with percentages varying slightly based on the antibiotic type and geographical distribution. Based on this meta-analysis, the overall *Klebsiella* resistance in Ethiopia was found to be 53.75% (95% CI: 48.35—58.94%). Similar findings were reported from meta-analysis study done in sub-Saharan Africa and Iran, where the resistance of *Klebsiella* for some antibiotics was greater than 50% [17, 20]. This indicates more than half of the isolated *Klebsiella* species were resistance to the most common antibacterial drugs. The possible reasons could be over/under dose of antimicrobial drugs in treating infectious diseases, and empiric use of antibiotics which would inevitably lead to emergence of resistance [24].

Antimicrobial resistance is becoming a public health challenge in the 21st century as a result of many factors including misuse of antimicrobials by health professional and patients, inadequate surveillance systems [7, 27]. Particularly, the emergence and spread of resistant strains of *Klebsiella* species are a considerable threat to public health [37]. For *Klebsiella*, resistance to antimicrobials is a normal evolutionary process, but the resistance rate is frequently aggravated by misuse of antibiotics [13, 38]. As per previous study, from the different species of *Klebsiella*, *Klebsiella pneumoniae* is becoming known for its resistance properties to most of the last-line antibiotics [8].

In this meta-analysis, the subgroup analysis of regional prevalence of *Klebsiella* indicated that the highest prevalence of *Klebsiella* resistance (64.39%) was estimated in SNNP, which is relatively higher than Tigray region (46.16%) (Fig. 4). The observed variation might be due to differences in study location, hospital setup, and antimicrobial utilization. The level of heterogeneity of the included studies was high, by random model methods (I^2^ = 90.55%; P < 0.01). This indicates that, the included studies have been done in different study areas, study periods, and study populations, which could have an effect on the heterogeneity of the studies.

The other subgroup analysis, based on the mechanism of action of antimicrobials, showed that *Klebsiella* exhibited higher resistance to antimetabolites (66.91%), which is actually represented by single drug (trimethoprim-sulfamethoxazole), followed by cell wall synthesis inhibitors (61.61%)(Fig. 5). In our finding, there was only one drug (trimethoprim-Sulfamethoxazole) from the antimetabolites tested for *Klebsiella* resistance, which might have led to the highest value of resistance group compared to the others. However, the highest resistance rate of *Klebsiella* was noted from individual antibiotics grouped under cell wall synthesis inhibitors including ampicillin (90.56%), amoxicillin (76.01%) and amoxicillin-clavulanic acid (56.92%).

Our finding was concordant with studies done in West Africa where *Klebsiella* was resistant to Ampicillin (92.5%) and amoxicillin-clavulanic acid (66.1%) [39], and a little bit lower *Klebsiella* resistance to ampicillin was reported from the three districts of Uganda (66.5%) [40] and India [1]. The higher resistance rate of *Klebsiella* species to ampicillin and amoxicillin could possibly be due to the fact that the bacteria naturally produces extended spectrum beta-lactamases which inactivate beta lactam` antibiotics (ESBLs) [11, 20]. Our finding showed almost similar pooled *Klebsiella* resistance to ceftriaxone (47.6%) with previous meta-analysis done on wound infection in which more than half of *Klebsiella pneumoniae* isolates exhibited resistance to ceftriaxone (57%) [23].

The findings of this study indicated that there is high magnitude of *Klebsiella* antimicrobial resistance in Ethiopia. The possible reasons could be limited infection surveillance programs, the lack of communication between physicians and microbiologists, lack of standardized criteria to determine drug resistant isolates, limited laboratory facilities, and poor sanitation [20]. The relatively high rates of drug resistant isolates of *Klebsiella* seen in this meta-analysis might have negative effects on public health which could cause difficulty in treating *Klebsiella pneumonia* associated infections since only fewer effective drugs are available for treating these highly drug-resistant strains [20]. Hence, functional infection control committee, applying infection prevention protocols, advocating rational prescribing habits, appropriate antimicrobial therapy, health education; and improvement of personal and environmental hygiene need to be applied to curb the resistance problem [13, 18, 20, 41].

Some limitations in our study should be acknowledged. The pooled resistance of *Klebsiella* was not calculated based on species basis as there was shortage of studies done on individual species. Many of the included studies describe *Klebsiella pneumonia*, but rarely for other species. Therefore, this meta-analysis should be seen in the context of such limitations.

## Conclusion

In this systematic review and meta-analysis, the pooled *Klebsiella* resistance was found to be considerably high (53.75%) to most of the essential antibiotics in Ethiopia. *Klebsiella* was highly resistant to ampicillin and amoxicillin but relatively lower to amikacin. Therefore, implementing proper antibiotic prescription policies and appropriate antimicrobial therapy could be the potential interventional strategies to address the emerging resistance of *Klebsiella* species.

### Electronic supplementary material

Below is the link to the electronic supplementary material.

Supplementary Material 1

Supplementary Material 2

## Data Availability

There is no remaining data and materials; all information is clearly presented in the main manuscript.

## Abbreviations

AML: Amoxicillin
AMC: Amoxicillin-clavulanic acid
AMP: Ampicillin
C: Chloramphenicol
CIP: Ciprofloxacin
GEN: Gentamicin
CRO: Ceftriaxone
F: Nitrofurantoin
NA: Nalidixic acid
NOR: Norfoxacin
SXT: Trimethoprim/Sulfamethoxazole
TE: Tetracycline
