# Supplementary material for: Antimicrobial resistance pattern of Klebsiella isolated from various clinical samples in Ethiopia: a systematic review and meta-analysis
Source: BMC Infect Dis. 2023 Oct 2;23:643. doi: 10.1186/s12879-023-08633-x (PMC10544621; doi:10.1186/s12879-023-08633-x)
Supplement: Supplementary file 1 — Supplementary Material 1 [file 12879_2023_8633_MOESM1_ESM.docx]

**S2 file: Modified Newcastle-Ottawa Scale (NOS)**

**Antimicrobial resistance pattern of *Klebsiella* isolated from various clinical samples in Ethiopia: a systematic review and meta-analysis**

Leake Gebremeskel^1^, Tewolde Teklu^1^, Gebremicheal Gebresilasie^1^, Kald Beshir Tuem^2^

**Table : Results of the critical appraisal of the included studies**

| Study (first author) | Study design | Selection | | | | Comparability | Outcome | | Quality Score  out of 7 |
| --- | --- | --- | --- | --- | --- | --- | --- | --- | --- |
|  |  | Representativeness of the sample | Sample size | Ascertainment of exposure | Non-respondents | The subjects in different outcome groups are comparable, based on the study design or analysis. Confounding factors are controlled. | Assessment of outcome | Was follow-up long enough for outcomes to occur |  |
| Abebaw etal., 2018 | RCS | 1 | 1 | 1 | 1 | 0 | 1 | 1 | 6 |
| Abebe etal.,2019 | RCS | 1 | 1 | 1 | 1 | 0 | 1 | 1 | 6 |
| Adhanom etal.,2019 | CS | 1 | 1 | 0 | 1 | 0 | 1 | 1 | 5 |
| Alemayehu etal.,2019 | CS | 1 | 1 | 1 | 1 | 0 | 1 | 1 | 6 |
| Amsalu etal.,2017 | RCS | 1 | 1 | 1 | 1 | 0 | 1 | 1 | 6 |
| Awoke etal.,2019 | CS | 1 | 1 | 1 | 1 | 1 | 1 | 1 | 7 |
| Beyene etal., 2019 | CS | 1 | 1 | 1 | 1 | 0 | 1 | 1 | 6 |
| Bitew etal., 2017 | CS | 1 | 1 | 1 | 1 | 1 | 1 | 1 | 7 |
| Dereje etal., 2017 | CS | 0 | 0 | 1 | 1 | 0 | 1 | 1 | 4 |
| Dessalegn etal., 2014 | CS | 0 | 0 | 1 | 1 | 0 | 1 | 1 | 4 |
| Duffa etal., 2018 | CS | 1 | 1 | 1 | 1 | 1 | 0 | 1 | 6 |
| Eshetie etal.,2015 | CS | 1 | 1 | 1 | 1 | 0 | 1 | 1 | 6 |
| Feleke etal.,2018 | CS | 1 | 1 | 1 | 1 | 0 | 0 | 1 | 5 |
| Gashaw etal.,2018 | CS | 1 | 1 | 1 | 1 | 1 | 1 | 1 | 7 |
| Gebremariam etal.,2019 | CS | 1 | 0 | 1 | 1 | 0 | 0 | 1 | 4 |
| Getahun etal., 2017 | CS | 0 | 0 | 1 | 1 | 0 | 1 | 1 | 4 |
| Godebo etal.,2013 | CS | 1 | 1 | 1 | 1 | 0 | 1 | 1 | 6 |
| Gutema etal., 2018 | CS | 1 | 1 | 1 | 1 | 1 | 0 | 1 | 6 |
| Hailu etal.,2016 | RCS | 1 | 1 | 1 | 1 | 1 | 1 | 1 | 7 |
| Legese etal.,2017 | CS | 1 | 1 | 1 | 1 | 1 | 1 | 1 | 7 |
| Mama etal.,2014 | CS | 1 | 1 | 1 | 1 | 1 | 0 | 1 | 6 |
| Mamuye 2016 | CS | 0 | 0 | 1 | 1 | 0 | 1 | 1 | 4 |
| Mengesha etal.,2014 | CS | 1 | 1 | 1 | 1 | 0 | 0 | 1 | 5 |
| Mitiku etal.,2018 | CS | 1 | 1 | 1 | 1 | 1 | 0 | 1 | 6 |
| Moges etal.,2019 | CS | 1 | 1 | 1 | 1 | 0 | 0 | 1 | 5 |
| Mohammed etal.,2017 | CS | 0 | 1 | 1 | 1 | 1 | 1 | 1 | 6 |
| Molla etal.,2019 | CS | 0 | 0 | 1 | 1 | 1 | 1 | 1 | 5 |
| Negussie etal., 2015 | Cs | 1 | 1 | 1 | 1 | 1 | 1 | 1 | 7 |
| Sahile etal., 2016 | CS | 1 | 1 | 1 | 1 | 0 | 1 | 1 | 6 |
| Sorsa etal., 2019 | CS | 1 | 1 | 1 | 1 | 1 | 0 | 1 | 6 |
| Tadesse etal., 2019 | CS | 1 | 1 | 1 | 1 | 0 | 1 | 1 | 6 |
| Tadesse etal.,2018 | CS | 1 | 1 | 1 | 1 | 1 | 1 | 1 | 7 |
| Teklu etal., 2019 | CS | 1 | 1 | 1 | 1 | 1 | 1 | 1 | 7 |
| Wasihun & Zemen, 2015 | CS | 1 | 1 | 1 | 1 | 1 | 0 | 1 | 6 |
| Worku etal.,2017 | CS | 0 | 1 | 1 | 1 | 1 | 1 | 1 | 6 |

This scale has been adapted from Wells *et al.,* (2009), “the Newcastle-Ottawa Scale (NOS) for assessing the quality of nonrandomized studies in meta-analyses”. We considered the comparability is controlled if the standard laboratory procedure is described in the paper. Since the outcome of the media is obtained after full growth of the bacterial strains, we consider the follow up time was enough for all studies. In our scale, we assigned assessment outcomes one star as the results are read from the experiment.
